# Population Trend of the World’s Monitored Seabirds, 1950-2010

**DOI:** 10.1371/journal.pone.0129342

**Published:** 2015-06-09

**Authors:** Michelle Paleczny, Edd Hammill, Vasiliki Karpouzi, Daniel Pauly

**Affiliations:** 1 University of British Columbia, Vancouver, British Columbia, Canada; 2 School of the Environment, University of Technology, Sydney, Ultimo, New South Wales, 2007, Australia; University of Toronto, CANADA

## Abstract

Seabird population changes are good indicators of long-term and large-scale change in marine ecosystems, and important because of their many impacts on marine ecosystems. We assessed the population trend of the world’s monitored seabirds (1950–2010) by compiling a global database of seabird population size records and applying multivariate autoregressive state-space (MARSS) modeling to estimate the overall population trend of the portion of the population with sufficient data (i.e., at least five records). This monitored population represented approximately 19% of the global seabird population. We found the monitored portion of the global seabird population to have declined overall by 69.7% between 1950 and 2010. This declining trend may reflect the global seabird population trend, given the large and apparently representative sample. Furthermore, the largest declines were observed in families containing wide-ranging pelagic species, suggesting that pan-global populations may be more at risk than shorter-ranging coastal populations.

## Introduction

Human activities such as fisheries and pollution are threatening the world’s marine ecosystems [1], causing changes to species abundance and distribution that alter ecosystem structure, function and resilience [2–4]. In response, increasing numbers of marine biologists and managers seek to achieve management measures allowing the persistence of healthy, productive and resilient ecosystems [5]. Such ecosystem-based management requires better understanding of ecosystems pre-disturbance, as baselines of harvested and/or otherwise impacted species such as fish, marine mammals, and seabirds have shifted from their historical levels [6–9].

Seabird population changes are good indicators of long-term and large-scale change in marine ecosystems because seabird populations are relatively well-monitored, their ecology allows them to integrate long-term and large-scale signals (they are long-lived, wide-ranging and forage at high trophic levels) [10–11], and their populations are strongly influenced by threats to marine and coastal ecosystems. These threats include entanglement in fishing gear, overfishing of food sources, climate change, pollution, disturbance, direct exploitation, development, energy production, and introduced species (predators such as rats and cats introduced to breeding islands that were historically free of land-based predators) [12]. Knowledge of changes in seabird populations is also inherently important because seabirds play important roles in island and marine ecosystem processes, function and resilience, by acting as predators, scavengers, cross-ecosystem nutrient subsidizers, and ecosystem engineers [2, 13–16].

Despite the global importance of seabirds, both to marine ecosystems and as indicators of marine ecosystem status, analysis of their population trends is typically limited to the relatively small spatial and temporal scales at which data are collected. The only global assessment of seabird population status, based on extinction risk as assessed by the IUCN Red List of Threatened Species, indicates that one third of seabird species are threatened with extinction, one half are known or suspected to be in decline, and at least four species are extinct [12].

To investigate overall patterns in the world’s seabird population data over an ecologically-relevant timeframe, we assembled a global database of seabird population size records and applied multivariate autoregressive state-space (MARSS) modeling to estimate the global trajectory of all seabird populations with sufficient data (i.e., at least five records of population size between 1950 and 2010).

## Materials and Methods

### Constructing a global database of seabird population data

We constructed a global database of available primarily English-language seabird population size records worldwide for the years spanning 1950–2010. We compiled data per population, defined as the breeding population of a species occurring on an island or stretch of coastline in which data were most commonly aggregated for reporting (i.e., a country or discrete sub-region of a large country such as a group of islands or a province). In total, we found data for 3213 breeding populations belonging to 324 seabird species (S1 Table) [17] reported in 357 coastal stretches (S2 Table).

We obtained data from primary sources including journal articles, books, and unpublished reports. We obtained population sizes as breeding pairs or total population; for comparison between the two, we converted records reported in breeding pairs to total population assuming that the population includes 30% non-breeders, a commonly assumed estimate for global seabird studies [18–20]. If a population size was reported as a range (e.g., 100–200 breeding pairs), we assumed the population size to be the geometric mean of the minimum and maximum records; the geometric mean is the square root of the product of a pair of values, and is applied in ecology to approximate central tendency [21]. For an example of the population database contents, see S3 Table. The database derived from our study is maintained by the *Sea Around Us* Project and will be made publicly available at www.seaaroundus.org.

### Estimating time-series for the monitored portion of the global seabird population using MARSS modeling

To estimate the overall global population trend from a large collection of time-series for different seabird populations, we required a model that could handle missing data and account for both observation and process error. We selected multivariate autoregressive state-space (MARSS) modeling because it estimates population size based on time-series containing missing data, and estimates observation error (difference between actual and observed population size) and process error (year to year variability in population growth) [22]. A MARSS model is described by the following equations, taken from Holmes *et al* 2012 [22]

$$x_{t}=x_{t-1}+u+w_{t},\;\;\;\text{where}\;\;w_{t}\sim \text{MVN}\left(0,{\text{Q}}_{t}\right)$$ (1)

$$y_{t}=x_{t}+a+v_{t},\;\text{where}\;\;v_{t}\sim \text{MVN}\left(0,{\text{R}}_{t}\right)$$ (2)

In the Eqs 1 and 2, *x* is a m*T matrix representing the state of the random variable *X*_*t*_ at each time *t*. The parameter *w* is a m*T matrix of the process errors at time *t*, with a mean 0 and covariance matrix *Q*_*t*_. Parameter *y* represents a n*T matrix of the observation, some of which may be missing. *v* is a n*T column vector of the non-process errors, the observation errors at *t* and multivariate and normal with mean 0, and covariance matrix R_*t*_. *u* and *a* are parameters. As each of the seabird populations was reproductively and often geographically isolated, we assumed they had independent random errors (diagonal Q matrix) and had different population parameters (*u* and *a*). As the population census data were collected by different groups utilizing different techniques (even within the same population), and some species are easier to detect than others, we stipulated independent observation variances for each population (diagonal and unequal R matrix). However, much of the variation in process error may be the result of environmental, rather than demographic stochasticity, meaning that populations may show similar trends and the assumption of independent process error is invalid. We therefore tested the assumptions of independence by running subsequent versions of the MARSS model where process and measurement errors were set as equal for each population. We used maximum likelihood with an Expectation-Maximum algorithm to estimate population trends as an auto-regressive stochastic process for all populations that had at least five records in their time-series (a prerequisite). These 513 of the total 3213 populations (S1 Table) are hereafter referred to as the monitored portion of the global seabird population. MARSS models provide an estimate of the population size in the years when no survey was conducted. Using this technique we were then able to obtain population estimates for all of the 513 monitored populations between the years 1950–2010. To represent the overall trajectory of monitored seabird populations, we summed the estimated number of seabirds, and their standard errors across all populations for each year, and plotted the trajectory. To understand how the fixed parameters (*u* and *a*) contributed to the population trajectory compared to the random variances (*Q* and *R*), we calculated the size of *Q* and *R* as a proportion of *u* and *a*.

### Interpreting the global significance of the estimated trend in the monitored portion of the population

In order for the trend estimated for the monitored population to be representative of the global population, the monitored sub-sample would have to be representative of the global population, so we would expect the taxonomic diversity and spatial distribution of the monitored populations to be diverse and reflective of the overall global population. To assess this, we compared the monitored populations (i.e., those 513 populations with >4 records) and the unmonitored populations (i.e., those 2696 populations with 1–4 records) the diversity and representativeness of taxa, marine regions, declining populations, and missing populations (i.e., populations with no records).

Where population size estimates were required to compare representativeness of these groupings, we estimated population size per grouping as the sum of the average population size records for all populations contained within the grouping (e.g., sum per family of the average population size estimates for all contained populations) (S1 Table). Given differing data availability between populations, accuracy of these estimates is higher in the monitored populations.

## Results

### Global seabird population data and time-series for the monitored portion of the population

**Fig 1:**
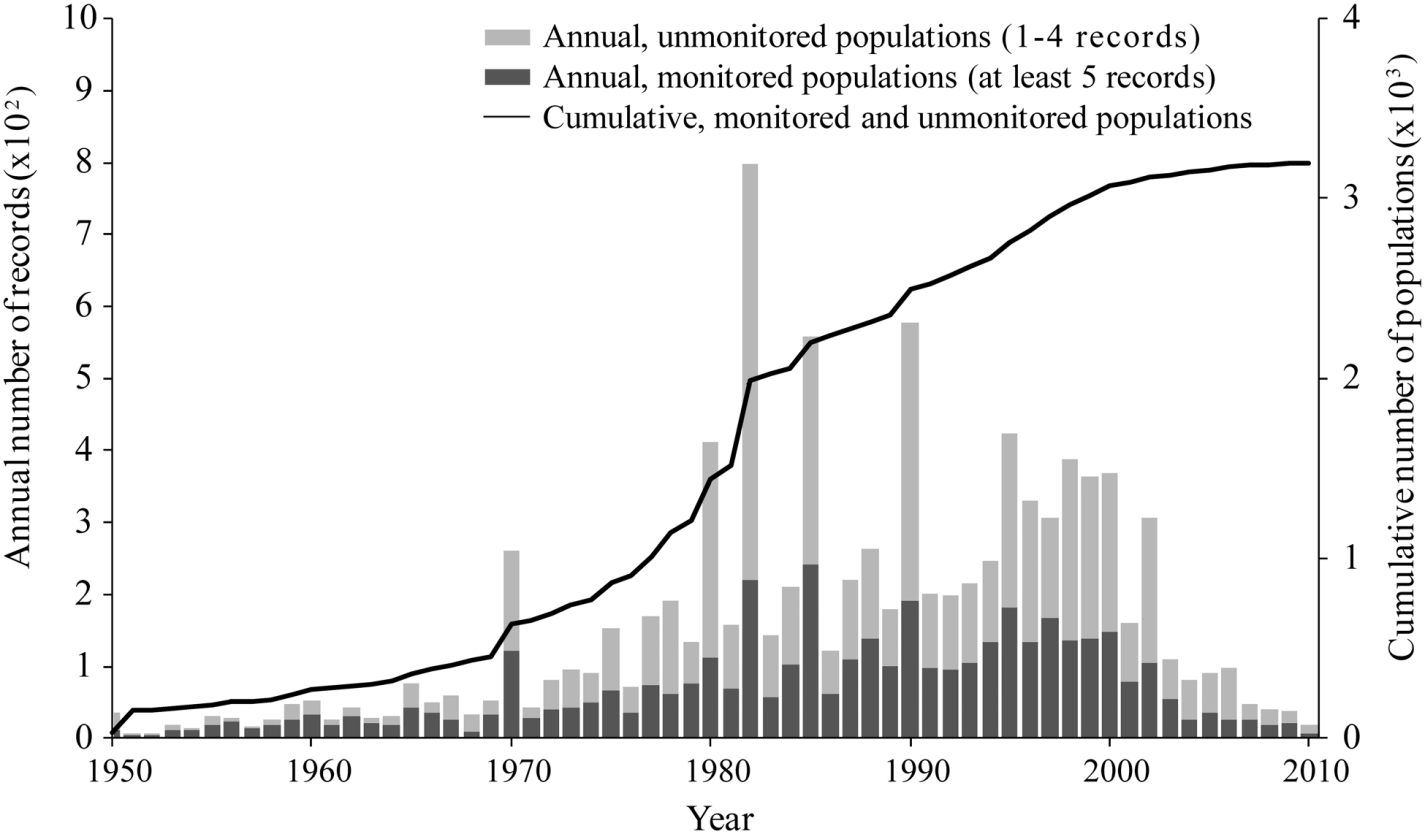
Number of records (annual and cumulative) in the global seabird population database, 1950–2010.

We compiled 9920 records for 3213 breeding populations; the number of records per population ranged from one to forty-nine and averaged three. The records were unevenly distributed throughout the decades and most numerous in the 1980s and 1990s (Fig 1).

**Fig 2:**
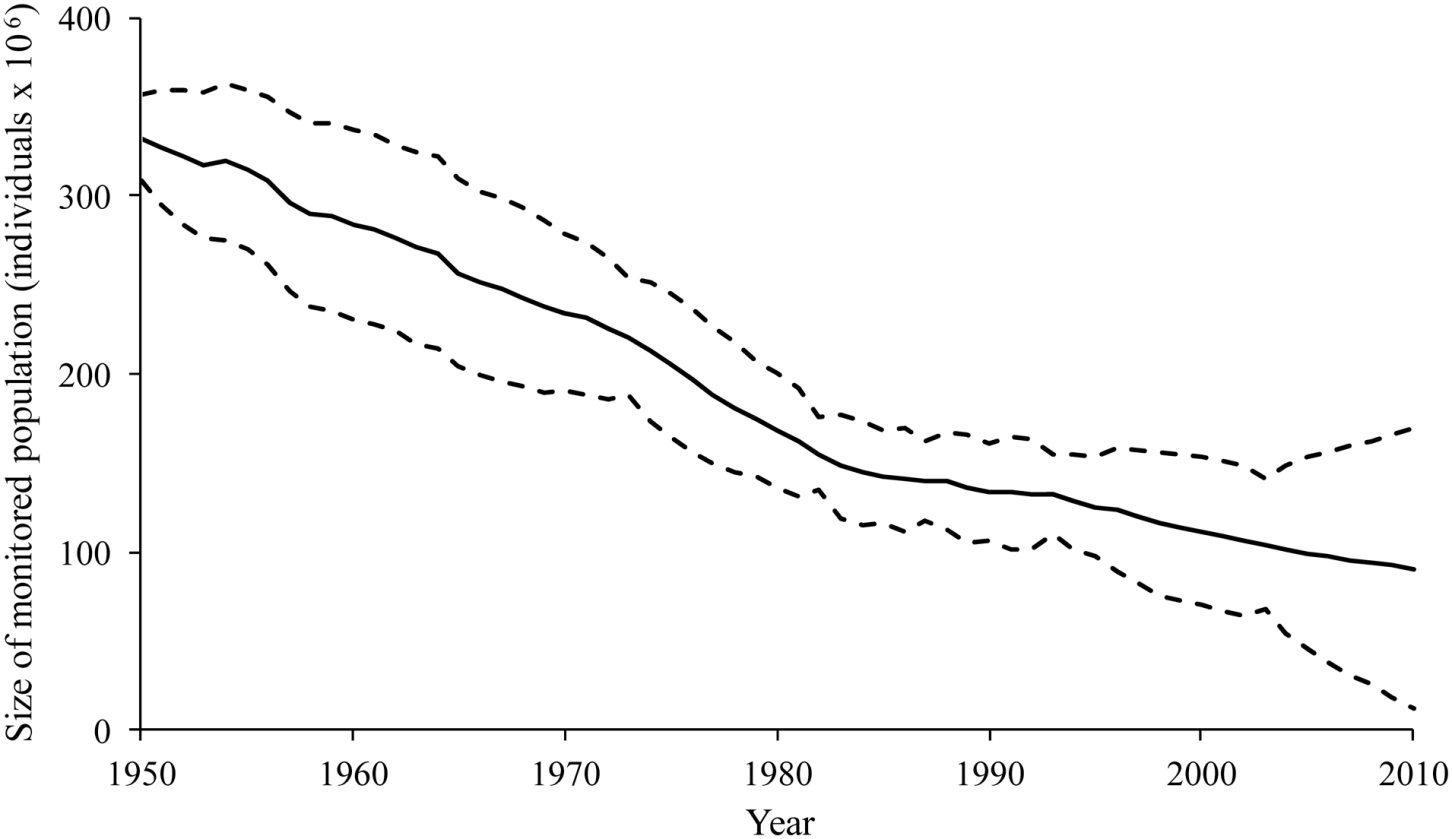
Population trend, 1950–2010, of the world’s monitored seabirds, estimated by multivariate autoregressive state-space (MARSS) modeling. Total number of birds in each year was calculated by summing the MARSS estimates for each population, including estimates from years without population estimates. The total population at the end of the time period was 30.3% of the population at the start, representing a 69.7% loss. Dashed lines represent 95% confidence intervals.

When Q and R matrices were set as diagonal and unequal (independent process and non-process errors used for each population), MARSS modeling revealed a substantial decline in seabird populations throughout the modern industrial era. This decline represented a 69.7% loss between 1950 and 2010, in the monitored portion of the global seabird population (Fig 2). The overall value of *u* across all populations was -8868.25, implying that on average, each seabird population lost a considerable number of individuals per year. A substantial proportion of this loss was due to large declines in the five most abundant populations, all located in the southern hemisphere. Between them, the populations of Sooty Terns (Sternidae) from French Polynesia and South Orkney, the South Sandwich Island populations of both Soft-plumaged Petrels and Kerguelen Petrels (both Procellariidae), and the Peruvian population of Guanay Cormorants (Phalacrocoracidae) accounted for over 30% of the total numbers of seabirds in the sampled population in 1950; all of these populations were reduced to less than 5% of their initial size by 2010.

When represented as proportions of the fixed effects, both process and non-process errors were considerably large (Table 1), indicating a substantial volume of random variation in the data. When either Q or R matrices were set as equal across populations and through time, the MARSS model failed to converge within 500 iterations, providing evidence that allowing these parameters to vary was the correct approach.

**Table 1:** Values of the fixed parameters of the model (*u* and *a*), as well as the process (*Q*) and non-process errors expressed as a proportion of the fixed effects.

Parameter	Value
*u* across populations	-8868.25
*a* across populaltions	1524.56
mean (*Q* as a proportion of *u*) across populations	0.531
mean (*R* as a proportion of *a*) across populations	0.638

For each population, process and non-process errors differed for each year of the sampling period. Within each population, we therefore used the mean of the process errors across years to produce the data in the table.

### Interpreting the global significance of the estimated trend in the monitored portion of the population

We assessed the diversity and representativeness of taxa, marine regions, and declining populations in the monitored portion of the population:

#### (i) Taxa

**Fig 3:**
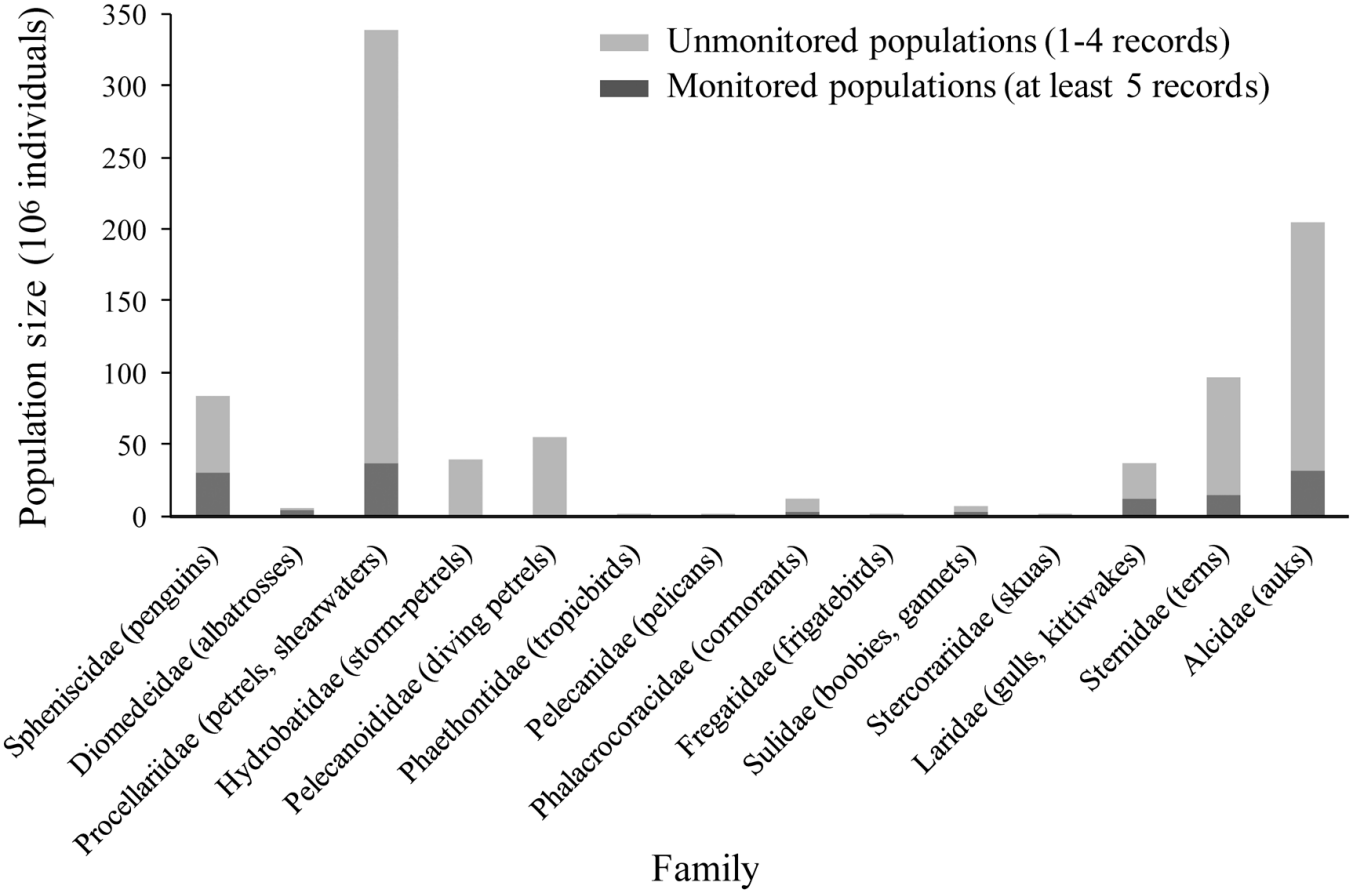
Population size per seabird family, monitored (i.e., ≥ 5 records) versus unmonitored (i.e., 1–4 records). Population size was estimated per population as the median between earliest and latest available records, then summed per family.

The taxonomic diversity of the monitored population was high, but not reflective of the taxonomic composition of the global seabird population. Half of all seabird species (i.e., 162 of 324) were represented in the monitored portion of the population (S1 Table). The extent to which each species was monitored varied; 17% of monitored species had 100% of their populations monitored, and 43% of species had at least 50% of their populations monitored. Thirteen families (i.e., all except Pelecanoididae) were represented in the monitored portion of the global seabird population (Fig 3). The extent to which each family was monitored varied from 1–5% (Hydrobatidae, Fregatidae, Phaethontidae, Stercorariidae), 12–20% (Procellariidae, Sternidae, Alcidae), 34–44% (Laridae, Sulidae), 54–59% (Phalacrocoracidae, Pelecanidae, Spheniscidae), and 89% (Diomedeidae). Of the seven numerically abundant seabird families that together account for approximately 97% of the global seabird population (Procellariidae, Alcidae, Sternidae, Spheniscidae, Pelecanoididae, Hydrobatidae, Laridae), two were poorly represented (Pelecanoididae and Hydrobatidae, jointly accounting for <1% of the monitored population) while the remaining five families were relatively well represented (jointly accounting for 92% of the monitored population). We found considerable differences in the population trajectories of different families over time. Of the monitored populations, we found overall increases in Alcidae (9.1%), Hydrobatidae (45.4%), and Sulidae (1.1%), and decreases in Diomedeidae (69.0%) Frigatidae (81.7%), Pelecanidae (35.3%), Laridae (17.4%), Phaethontidae (25.8%), Phalacrocoracidae (73.6%), Procellariidae (79.6%), Spheniscidae (32.3%), Stercorariidae (65.4%), Sternidae (85.8%).

#### (ii) Marine regions

**Fig 4:**
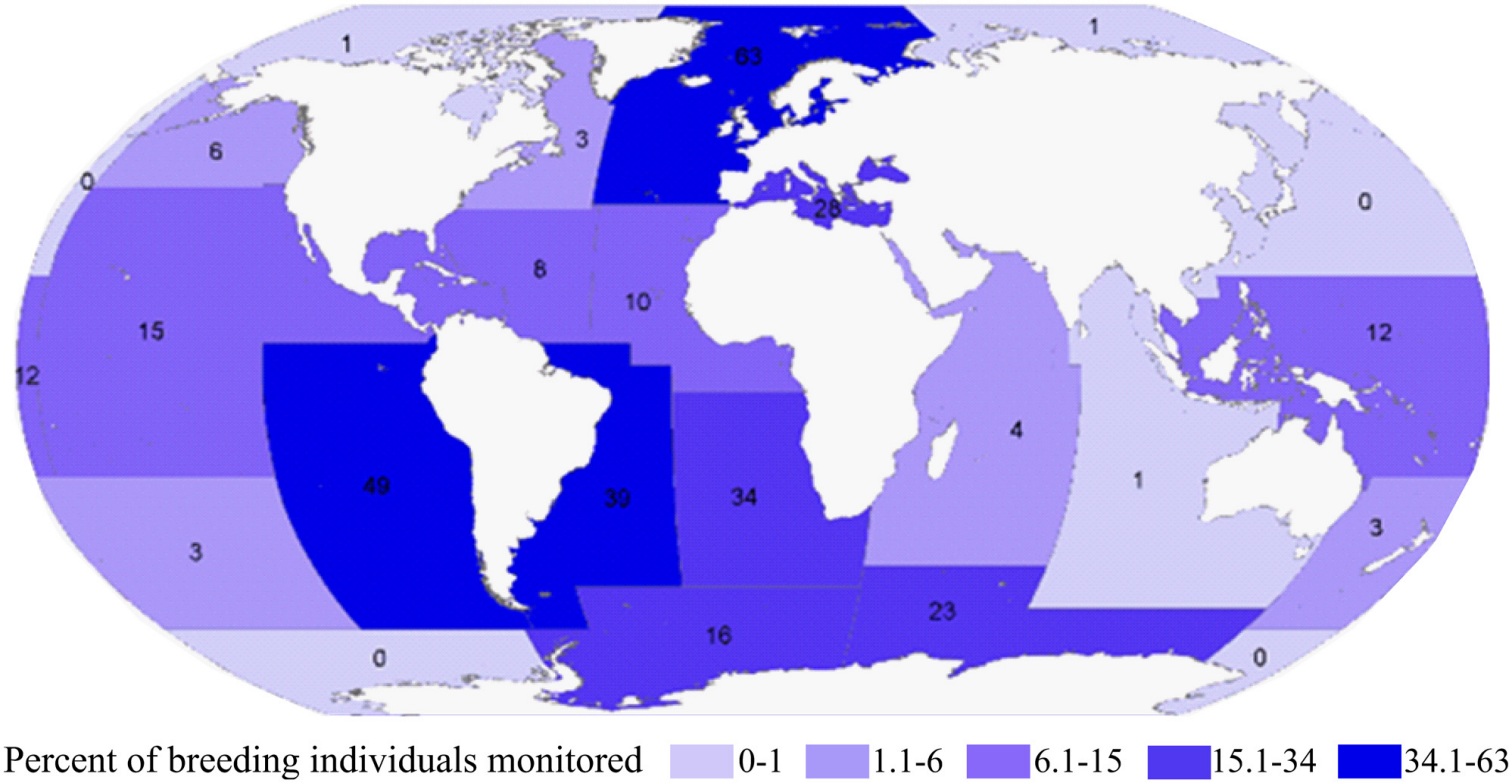
Percent of breeding individuals monitored, per marine region.

The spatial distribution of records was heterogeneous and not necessarily reflective of seabird numerical abundance (Fig 4). Marine regions that were relatively well-represented included the southeast Pacific (49% of breeding population monitored), southwest Atlantic (39% of breeding population monitored), and northeast Atlantic (63% of breeding population monitored). Marine regions that were relatively poorly represented included the northwest Pacific (0% of breeding population monitored), northwest Atlantic (3% of the breeding population monitored), southwest Pacific (3% of the breeding population monitored), Indian Ocean (<4% of the breeding population monitored), and polar regions (in some parts 0% of the breeding population monitored).

#### (iii) Declining populations

The monitored population, in comparison with the unmonitored population, contained a slightly higher percentage of declining populations, but also a higher percentage of increasing populations. Based on available data and methods described above, overall decline was detected in 38% of the monitored populations and 27% of the unmonitored populations, increase was detected in 61% of monitored populations and 29% of unmonitored populations, and no change was detected in 1% of monitored populations and 44% of unmonitored populations (the latter is due to the high number of populations with only one population record). Threatened species (i.e., status of Vulnerable, Endangered or Critically Endangered on the International Union for Conservation of Nature (IUCN) Red List) comprised 26% of monitored species; this percentage is slightly lower than that across all of the world’s species (i.e., 28%) [12].

#### (iv) Missing populations

The number missing populations (i.e., with no records) is unknown, but the proportion of the global population that they represent appears to be low; Using methods described above, we estimate the sum of the monitored and unmonitored population sizes to be 0.77 billion individuals from 324 species, which is comparable to a more coarse (i.e., family-scale) estimate of global seabird population size of 0.7 billion individuals from 309 species [20].

## Discussion

### Drivers of a declining trend in the monitored portion of the global seabird population

The cause of the estimated overall decline in seabird populations is likely a suite of threatening human activities—introduced species at nesting colonies (e.g., rats, cats), entanglement in fishing gear at sea, overfishing of food sources by humans, climate change and severe weather, pollution, disturbance, direct exploitation (harvesting chicks, eggs, adults), development, and energy production [12]. Some of these threats affect most species across the globe (e.g., climate change is a global process, although some regions will be more detrimentally affected), while others will be more local processes, or impact species disproportionately based on their ecology and/or life-history attributes (e.g., overfishing of food sources has a strong negative impact on seabirds with small body size, specialized diets, energetically expensive foraging, and surface foraging [23]). The impacts of some threats pre-date our seabird population data [24–27] and therefore a potentially large percentage of seabird populations were already suppressed prior to our 1950 baseline. Additionally, exploitation of other marine taxa (e.g., whales, sea otters, fish) has contributed indirectly to changes in seabird populations by altering the quantity, quality and spatial distribution of their prey [28]. While seabird conservation efforts have been successful in reducing mortality of some species in recent decades, for example, banning some direct exploitation [27], eradicating some introduced predators [29], reducing some entanglement in fishing gear [30], these efforts are evidently not sufficient in terms of stopping or reversing large-scale seabird decline, especially as new threats emerge, notably industrial harvesting to food [31]. Throughout the 1950–2010 timeframe of our study, overall threat rates have likely increased alongside growth in human population and industrialization although there may be annual variation in threats and threat response. Relatively low variation was observed in the rate of decline of the monitored population of seabirds throughout the timeframe (Fig 2), perhaps due to the continuous growth and expansion of threats and/or the lack of temporal resolution in available data. Climate oscillations can contribute to patterns in seabird abundance by affecting prey availability [32], although they are not likely the driving cause of the observed decline, as the continuous decline of seabirds over the timeframe of this study does not mirror any known climate oscillations. The interaction of climate cycles with threatening human activities may however be at play; in both Peru and California, depletion of seabird prey by fisheries has contributed to decreased resilience of seabirds to natural climate cycles, resulting in declines under poor climatic conditions and lack of natural rebound under good climatic conditions [33–34].

### Global significance of the estimated trend in the monitored portion of the global population

Although the monitored portion of the world’s seabirds represents a subsample of the global seabird population, and we therefore cannot be certain the decline we have uncovered represents the pattern for the global seabird population, our relatively large sample suggests that a substantial global decline has occurred. Our findings also correspond with the highly threatened status of many of the world’s seabird species [12].

Investigation of how well our subsample represents the global population revealed no strong bias towards particular taxa, marine regions or declining populations. Half of all seabird species were represented and thirteen of fourteen families were represented. While families were not sampled in proportion to their numerical abundance, there was no apparent bias in sampling related to the proportion of threatened species per family [12]. For example, particularly well-represented families (in absolute and relative terms) include both families containing relatively high percentages of threatened species (Spheniscidae, Procellariidae, Diomedeidae) and relatively low percentages of threatened species (Alcidae, Laridae), while two families with particularly high percentages of threatened species are poorly represented (Hydrobatidae, Pelecanoididae) [12]. Sampling effort was not even across marine regions, but there was no apparent relationship between sampling effort and overall threat intensity across marine regions; of the regions with strongest threatening human activities, some were relatively well-monitored (northeast Atlantic) while others were relatively poorly monitored (Asia) [1]. While the monitored portion in comparison with the unmonitored portion of the global population contained a slightly higher percentage of declining populations, it also contained a much higher percentage of increasing populations (because the unmonitored population had a high number of populations with only one record), and a slightly lower representation of threatened species. A seabird global population estimate derived from our data was similar to an existing global estimate, suggesting that only a small minority of the global seabird population lacks some degree of population monitoring during the timeframe of the study. Overall, the lack of bias observed in the taxa, marine regions, and declining populations in the subsample, in addition to indication that most of the world’s population has been sampled at least once, suggests that the trend observed for the monitored portion of the population may reflect an overall trend in the global seabird population.

### Underlying population trends in the monitored portion of the global seabird population

Of the 3213 populations of seabirds included in this analysis, a 61% of the monitored populations and 27% of the unmonitored populations were observed to increase. However, within the monitored population we observed a 69.7% decrease in overall seabird numbers. This discrepancy between the large number of populations observed to increase, and the overall decrease in total numbers stems from the fact that the populations showing increases were relatively small. Conversely, a substantial proportion of the overall loss was due to large declines in the five most abundant populations, all located in the southern hemisphere. Generally, the populations that were observed to increase tended to be small coastal populations, which are more likely to be partitioned into smaller units due to the ease of assigning geographical boundaries. Whereas the populations that were observed to decline were generally pelagic species that may contain many more individuals and for whom assigning geographic boundaries is difficult. Further support for the relationship between coastal or pelagic and likelihood of decline can be seen in the family level differences in rates of decline. The family undergoing the greatest decline (Sternidae) contains species that undertake the long migrations and have pan global distributions. Generally, other families that have large home ranges (Stercorariidae, Diomedeidae) were observed to undergo substantial declines. In addition to differences in separating populations of pelagic species, smaller, coastal populations may react better to conservation actions. Undertaking conservation and restorative actions for small populations may be more feasible, as fewer governing bodies are likely to be involved and the geographic area is smaller. For example, the removal of cats and rats from small islands has been achieved on multiple occasions and been shown to increase local seabird numbers [35], however undertaking conservation actions for pan-global populations, such as reducing oceanic pollution or lowering fishing pressure will be considerably more challenging. Therefore, while many small populations have been observed to increase, these increases are swamped by large decreases in fewer, but larger populations.

## Conclusion

Our analysis revealed long-term and large-scale population decline, by 69.7% between 1950 and 2010, in the world’s monitored seabirds (i.e., populations monitored at least five times, representing approximately 19% of the global seabird population). This declining trend may reflect the global seabird population trend, given the large and apparently representative sample. Furthermore, the largest declines were observed in families containing wide-ranging pelagic species, suggesting that pan-global populations may be more at risk than shorter-ranging coastal populations.

## Supporting Information

S1 Table Seabird species considered in this study (species represented in the monitored population are in bold). (DOCX)

S2 Table List of coastal stretches for which breeding population data were available. (DOCX)

S3 Table Example of contents of the population size database for the Blue Petrel (*Halobaena caerulea*). (DOCX)
