# Supplementary material for: Population Trend of the World’s Monitored Seabirds, 1950-2010
Source: PLoS One. 2015 Jun 9;10(6):e0129342. doi: 10.1371/journal.pone.0129342 (PMC4461279; doi:10.1371/journal.pone.0129342)
Supplement: S1 Table — (DOCX) [file pone.0129342.s001.docx]

Supplementary online materials to

Population trend of the world’s monitored seabirds, 1950-2010

by

Michelle Paleczny^1¶^*, Edd Hammill^1,2¶^,Vasiliki Karpouzi^1^, Daniel Pauly^1^

^1^ University of British Columbia, Vancouver, British Columbia, Canada

^2^ School of the Environment, University of Technology, Sydney, Ultimo, New South Wales 2007, Australia

¶ The first and second authors contributed equally to this work.

*Correspondence to: e-mail: m.paleczny@fisheries.ubc.ca

**S1 Table. Seabird species considered in this study (species represented in the monitored population are in bold).**

| **Family** | **Species (common)** | ***Species (latin)*** |
| --- | --- | --- |
| Sulidae | **Abbott's Booby** | ***Sula abbotti*** |
|  | **Australasian Gannet** | ***Morus serrator*** |
|  | Blue-footed Booby | *Sula nebouxii* |
|  | **Brown Booby** | ***Sula leucogaster*** |
|  | **Cape Gannet** | ***Morus capensis*** |
|  | **Masked Booby** | ***Sula dactylatra*** |
|  | **Nazca Booby** | ***Sula granti*** |
|  | **Northern Gannet** | ***Morus bassanus*** |
|  | **Peruvian Booby** | ***Sula variegata*** |
|  | **Red-footed Booby** | ***Sula sula*** |
| Spheniscidae | Adelie Penguin | *Pygoscelis adeliae* |
|  | Blue Penguin | *Eudyptula minor* |
|  | **Chinstrap Penguin** | *Pygoscelis antarctica* |
|  | **Emperor Penguin** | *Aptenodytes forsteri* |
|  | Erect-crested Penguin | *Eudyptes sclateri* |
|  | Fiordland Penguin | *Eudyptes pachyrhynchus* |
|  | **Galapagos Penguin** | *Spheniscus mendiculus* |
|  | **Gentoo Penguin** | *Pygoscelis papua* |
|  | Humboldt Penguin | *Spheniscus humboldti* |
|  | **Jackass Penguin** | *Spheniscus demersus* |
|  | **King Penguin** | *Aptenodytes patagonicus* |
|  | **Macaroni Penguin** | *Eudyptes chrysolophus* |
|  | **Magellanic Penguin** | *Spheniscus magellanicus* |
|  | **Rockhopper Penguin** | *Eudyptes chrysocome* |
|  | Royal Penguin | *Eudyptes schlegeli* |
|  | **Snares Penguin** | *Eudyptes robustus* |
|  | Yellow-eyed Penguin | *Megadyptes antipodes* |
| Diomedeidae | **Amsterdam Albatross** | ***Diomedea amsterdamensis*** |
|  | **Antipodean Albatross** | ***Diomedea antipodensis*** |
|  | **Black-browed Albatross** | ***Thalassarche melanophris*** |
|  | **Black-footed Albatross** | ***Phoebastria nigripes*** |
|  | **Buller's Albatross** | ***Thalassarche bulleri*** |
|  | **Campbell Albatross** | ***Thalassarche impavida*** |
|  | **Chatham Albatross** | ***Thalassarche eremita*** |
|  | **Gibson's Albatross** | ***Diomedea gibsoni*** |
|  | **Grey-headed Albatross** | ***Thalassarche chrysostoma*** |
|  | **Indian Yellow-nosed Albatross** | ***Thalassarche carteri*** |
|  | **Laysan Albatross** | ***Phoebastria immutabilis*** |
|  | **Light-mantled Albatross** | ***Northern Royal Albatross*** |
|  | Northern Royal Albatross | *Diomedea sanfordi* |
|  | Salvin's Albatross | *Thalassarche salvini* |
|  | **Short-tailed Albatross** | ***Phoebastria albatrus*** |
|  | **Shy Albatross** | ***Thalassarche cauta*** |
|  | **Sooty Albatross** | ***Phoebetria fusca*** |
|  | **Southern Royal Albatross** | ***Diomedea epomophora*** |
|  | **Tristan Albatross** | ***Diomedea dabbenena*** |
|  | **Wandering Albatross** | ***Diomedea exulans*** |
|  | **Waved Albatross** | ***Phoebastria irrorata*** |
|  | **Yellow-nosed Albatross** | ***Thalassarche chlororhynchos*** |
| Procellariidae | Blue Petrel | *Halobaena caerulea* |
|  | Antarctic Prion | *Pachyptila desolata* |
|  | **Antarctic Petrel** | ***Thalassoica antarctica*** |
|  | Atlantic Petrel | *Pterodroma incerta* |
|  | Audubon's Shearwater | *Puffinus lherminieri* |
|  | Balearic Shearwater | *Puffinus mauretanicus* |
|  | **Barau's Petrel** | ***Pterodroma baraui*** |
|  | Beck's Petrel | *Pseudobulweria becki* |
|  | **Bermuda Petrel** | ***Pterodroma cahow*** |
|  | Black-capped Petrel | *Pterodroma hasitata* |
|  | **Black-vented Shearwater** | ***Puffinus opisthomelas*** |
|  | Black-winged Petrel | *Pterodroma nigripennis* |
|  | Bonin Petrel | *Pterodroma hypoleuca* |
|  | Broad-billed Prion | *Pachyptila vittata* |
|  | Buller's Shearwater | *Puffinus bulleri* |
|  | **Bulwer's Petrel** | ***Bulweria bulwerii*** |
|  | **Cape Petrel** | ***Daption capense*** |
|  | **Cape Verde Petrel** | ***Pterodroma feae*** |
|  | Cape Verde Shearwater | *Calonectris edwardsii* |
|  | **Chatham Island Petrel** | ***Pterodroma axillaris*** |
|  | **Christmas Shearwater** | ***Puffinus nativitatis*** |
|  | Collared Petrel | *Pterodroma brevipes* |
|  | Cook's Petrel | *Pterodroma cookii* |
|  | **Cory's Shearwater** | ***Calonectris diomedea*** |
|  | De Filippi's Petrel | *Pterodroma defilippiana* |
|  | Fairy Prion | *Pachyptila turtur* |
|  | Fiji Petrel | *Pseudobulweria macgillivrayi* |
|  | Flesh-footed Shearwater | *Puffinus carneipes* |
|  | Fluttering Shearwater | *Puffinus gavia* |
|  | Fulmar Prion | *Pachyptila crassirostris* |
|  | **Galapagos Petrel** | ***Pterodroma phaeopygia*** |
|  | **Gould's Petrel** | ***Pterodroma leucoptera*** |
|  | **Greater Shearwater** | ***Puffinus gravis*** |
|  | Great-winged Petrel | *Pterodroma macroptera* |
|  | Grey Petrel | *Procellaria cinerea* |
|  | **Hawaiian Dark-rumped Petrel** | ***Pterodroma sandvicensis*** |
|  | Heinroth's Shearwater | *Puffinus heinrothi* |
|  | Henderson Petrel | *Pterodroma atrata* |
|  | Herald Petrel | *Pterodroma heraldica* |
|  | **Hutton's Shearwater** | ***Puffinus huttoni*** |
|  | Jamaica Petrel | *Pterodroma caribbaea* |
|  | Jouanin's Petrel | *Bulweria fallax* |
|  | Juan Fernandez Petrel | *Pterodroma externa* |
|  | Kerguelen Petrel | *Lugensa brevirostris* |
|  | Kermadec Petrel | *Pterodroma neglecta* |
|  | **Levantine Shearwater** | ***Puffinus yelkouan*** |
|  | **Little Shearwater** | ***Puffinus assimilis*** |
|  | **Madeira Petrel** | ***Pterodroma madeira*** |
|  | Magenta Petrel | *Pterodroma magentae* |
|  | **Manx Shearwater** | ***Puffinus puffinus*** |
|  | Mascarene Petrel | *Pseudobulweria aterrima* |
|  | Mottled Petrel | *Pterodroma inexpectata* |
|  | Murphy's Petrel | *Pterodroma ultima* |
|  | Newell's Shearwater | *Puffinus newelli* |
|  | **Northern Fulmar** | ***Fulmarus glacialis*** |
|  | **Northern Giant Petrel** | ***Macronectes halli*** |
|  | Parkinson's Petrel | *Procellaria parkinsoni* |
|  | Phoenix Petrel | *Pterodroma alba* |
|  | Pink-footed Shearwater | *Puffinus creatopus* |
|  | Providence Petrel | *Pterodroma solandri* |
|  | Pycroft's Petrel | *Pterodroma pycrofti* |
|  | **Salvin's Prion** | ***Pachyptila salvini*** |
|  | Short-tailed Shearwater | *Puffinus tenuirostris* |
|  | **Snow Petrel** | ***Pagodroma nivea*** |
|  | **Soft-plumaged Petrel** | ***Pterodroma mollis*** |
|  | Sooty Shearwater | *Puffinus griseus* |
|  | Southern Fulmar | *Fulmarus glacialoides* |
|  | **Southern Giant Petrel** | ***Macronectes giganteus*** |
|  | Spectacled Petrel | *Procellaria conspicillata* |
|  | Stejneger's Petrel | *Pterodroma longirostris* |
|  | Streaked Shearwater | *Calonectris leucomelas* |
|  | Tahiti Petrel | *Pseudobulweria rostrata* |
|  | Thin-billed Prion | *Pachyptila belcheri* |
|  | Townsend's Shearwater | *Puffinus auricularis* |
|  | Trindade Petrel | *Pterodroma arminjoniana* |
|  | **Wedge-tailed Shearwater** | ***Puffinus pacificus*** |
|  | **Westland Petrel** | ***Procellaria westlandica*** |
|  | **White-chinned Petrel** | ***Procellaria aequinoctialis*** |
|  | White-headed Petrel | *Pterodroma lessonii* |
|  | White-necked Petrel | *Pterodroma cervicalis* |
| Hydrobatidae | Ashy Storm Petrel | *Oceanodroma homochroa* |
|  | Black Storm Petrel | *Oceanodroma melania* |
|  | Black-bellied Storm Petrel | *Black-bellied Storm Petrel* |
|  | **European Storm Petrel** | ***Hydrobates pelagicus*** |
|  | Forked-tailed Storm Petrel | *Oceanodroma furcata* |
|  | Grey-backed Storm Petrel | *Garrodia nereis* |
|  | Hornby's Storm Petrel | *Oceanodroma hornbyi* |
|  | **Leach's Storm Petrel** | ***Oceanodroma leucorhoa*** |
|  | Least Storm Petrel | *Halocyptena microsoma* |
|  | **Madeiran Storm Petrel** | ***Oceanodroma castro*** |
|  | Markham's Storm Petrel | *Oceanodroma markhami* |
|  | Matsudaira's Storm Petrel | *Oceanodroma matsudairae* |
|  | Swinhoe's Storm Petrel | *Oceanodroma monorhis* |
|  | Tristram's Storm Petrel | *Oceanodroma tristrami* |
|  | Wedge-rumped Storm Petrel | *Oceanodroma tethys* |
|  | White-bellied Storm Petrel | *Fregetta grallaria* |
|  | White-faced Storm Petrel | *Pelagodroma marina* |
|  | White-throated Storm Petrel | *Nesocarbo fuliginosa* |
|  | White-vented Storm Petrel | *Oceanites gracilis* |
|  | Wilson's Storm Petrel | *Oceanites oceanicus* |
| Pelecanoididae | Common Diving Petrel | *Pelecanoides urinatrix* |
|  | Magellanic Diving Petrel | *Pelecanoides magellanicus* |
|  | Peruvian Diving Petrel | *Pelecanoides garnotii* |
|  | South Georgia Diving Petrel | *Pelecanoides georgicus* |
| Phaethontidae | **Red-billed Tropicbird** | ***Phaethon aethereus*** |
|  | **Red-tailed Tropicbird** | ***Phaethon rubricauda*** |
|  | **White-tailed Tropicbird** | ***Phaethon lepturus*** |
| Pelecanidae | American White Pelican | *Pelecanus erythrorhynchos* |
|  | Australian Pelican | *Pelecanus conspicillatus* |
|  | **Brown Pelican** | ***Pelecanus occidentalis*** |
|  | **Dalmatian Pelican** | ***Pelecanus crispus*** |
|  | **Great White Pelican** | ***Pelecanus onocrotalus*** |
|  | **Peruvian Pelican** | ***Pelecanus thagus*** |
|  | **Pink-backed Pelican** | ***Pelecanus rufescens*** |
|  | **Spot-billed Pelican** | ***Pelecanus philippensis*** |
| Phalacrocoracidae | **Antarctic Shag** | ***Notocarbo bransfieldensis*** |
|  | Auckland Island Shag | *Euleucocarbo colensoi* |
|  | **Bank Cormorant** | ***Compsohalieus neglectus*** |
|  | Black-faced Cormorant | *Compsohalieus fuscescens* |
|  | Bounty Island Shag | *Euleucocarbo ranfurlyi* |
|  | **Brandt's Cormorant** | ***Compsohalieus penicillatus*** |
|  | Campbell Shag | *Nesocarbo campbelli* |
|  | **Cape Cormorant** | ***Leucocarbo capensis*** |
|  | **Chatham Island Shag** | ***Euleucocarbo onslowi*** |
|  | **Crowned Cormorant** | ***Microcarbo coronatus*** |
|  | **Double-crested Cormorant** | ***Hypoleucos auritus*** |
|  | **European Shag** | ***Phalacrocorax aristotelis*** |
|  | **Flightless Cormorant** | ***Compsohalieus harrisi*** |
|  | **Great Cormorant** | ***Phalacrocorax carbo*** |
|  | **Guanay Cormorant** | ***Leucocarbo bougainvillii*** |
|  | Imperial Shag | *Notocarbo atriceps* |
|  | **Indian Cormorant** | ***Hypoleucos fuscicollis*** |
|  | Japanese Cormorant | *Phalacrocorax capillatus* |
|  | Kerguelen Shag | *Notocarbo verrucosus* |
|  | King Cormorant | *Notocarbo albiventer* |
|  | Little Black Cormorant | *Hypoleucos sulcirostris* |
|  | **Little Cormorant** | ***Microcarbo niger*** |
|  | Little Pied Cormorant | *Microcarbo melanoleucos* |
|  | **Long-tailed Cormorant** | ***Microcarbo africanus*** |
|  | Macquarie Shag | *Phalacrocorax purpurascens* |
|  | Neotropic Cormorant | *Hypoleucos brasiliensis* |
|  | New Zealand King Shag | *Euleucocarbo carunculatus* |
|  | **Pelagic Cormorant** | ***Strictocarbo pelagicus*** |
|  | **Pied Cormorant** | ***Hypoleucos varius*** |
|  | Pitt Island Shag | *Strictocarbo featherstoni* |
|  | **Pygmy Cormorant** | ***Microcarbo pygmaeus*** |
|  | Red-faced Cormorant | *Strictocarbo urile* |
|  | Red-legged Cormorant | *Strictocarbo gaimardi* |
|  | Rock Cormorant | *Strictocarbo magellanicus* |
|  | Socotra Cormorant | *Leucocarbo nigrogularis* |
|  | South Georgia Shag | *Notocarbo georgianus* |
|  | Spotted Shag | *Strictocarbo punctatus* |
|  | Stewart Island Shag | *Euleucocarbo chalconotus* |
|  | **Ascension Frigatebird** | ***Fregata aquila*** |
|  | Christmas Island Frigatebird | *Fregata andrewsi* |
|  | Great Frigatebird | *Fregata minor* |
|  | Lesser Frigatebird | *Fregata ariel* |
|  | **Magnificent Frigatebird** | ***Fregata magnificens*** |
| Stercorariidae | **Brown Skua** | ***Catharacta antarctica*** |
|  | Chilean Skua | *Catharacta chilensis* |
|  | **Great Skua** | ***Catharacta skua*** |
|  | Long-tailed Jaeger | *Stercorarius longicaudus* |
|  | **Parasitic Jaeger** | ***Stercorarius parasiticus*** |
|  | Pomarine Jaeger | *Catharacta pomarinus* |
|  | **South Polar Skua** | ***Catharacta maccormicki*** |
| Laridae | Armenian Gull | *Larus armenicus* |
|  | **Audouin's Gull** | ***Larus audouini*** |
|  | Band-tailed Gull | *Larus belcheri* |
|  | Black-billed Gull | *Larus bulleri* |
|  | **Black-legged Kittiwake** | ***Rissa tridactyla*** |
|  | **Black-tailed Gull** | ***Larus crassirostris*** |
|  | Bonaparte's Gull | *Larus philadelphia* |
|  | Brown-hooded Gull | *Larus maculipennis* |
|  | **California Gull** | ***Larus californicus*** |
|  | **Common Black-headed Gull** | ***Larus ridibundus*** |
|  | **Common Gull** | ***Larus canus*** |
|  | Dolphin Gull | *Larus scoresbii* |
|  | **Glaucous Gull** | ***Larus hyperboreus*** |
|  | **Glaucous-winged Gull** | ***Larus glaucescens*** |
|  | Gray Gull | *Larus modestus* |
|  | **Great Black-backed Gull** | ***Larus marinus*** |
|  | Great Black-headed Gull | *Larus ichthyaetus* |
|  | **Grey-headed Gull** | ***Larus cirrocephalus*** |
|  | **Hartlaub's Gull** | ***Larus hartlaubii*** |
|  | Heermann's Gull | *Larus heermanni* |
|  | **Herring Gull** | ***Larus argentatus*** |
|  | Iceland Gull | *Larus glaucoides* |
|  | **Ivory Gull** | ***Pagophila eburnea*** |
|  | **Kelp Gull** | ***Larus dominicanus*** |
|  | **Laughing Gull** | ***Larus atricilla*** |
|  | Lava Gull | *Larus fuliginosus* |
|  | **Lesser Black-backed Gull** | ***Larus fuscus*** |
|  | **Little Gull** | ***Larus minutus*** |
|  | **Mediterranean Gull** | ***Larus melanocephalus*** |
|  | Olrog's Gull | *Larus atlanticus* |
|  | Pacific Gull | *Larus pacificus* |
|  | **Red-billed Gull** | ***Larus scopulinus*** |
|  | **Red-legged Kittiwake** | ***Rissa brevirostris*** |
|  | **Ring-billed Gull** | ***Larus delawarensis*** |
|  | Ross's Gull | *Rhodostethia rosea* |
|  | Sabine's Gull | *Xema sabini* |
|  | Saunder's Gull | *Larus saundersi* |
|  | Silver Gull | *Larus novaehollandiae* |
|  | Slaty-backed Gull | *Larus schistisagus* |
|  | **Slender-Billed Gull** | ***Larus genei*** |
|  | Sooty Gull | *Larus hemprichi* |
|  | Swallow-tailed Gull | *Creagrus furcatus* |
|  | Thayer's Gull | *Larus thayeri* |
|  | **Western Gull** | ***Larus occidentalis*** |
|  | White-eyed Gull | *Larus leucophthalmus* |
|  | Yellow-footed Gull | *Larus livens* |
|  | **Yellow-legged Gull** | ***Larus cachinnans*** |
| Sternidae | **Sooty Tern** | ***Sterna fuscata*** |
|  | Aleutian Tern | *Sterna aleutica* |
|  | **Antarctic Tern** | ***Sterna vittata*** |
|  | **Arctic Tern** | ***Sterna paradisaea*** |
|  | **Black Noddy** | ***Anous minutus*** |
|  | Black Tern | *Chlidonias niger* |
|  | **Black-bellied Tern** | ***Sterna acuticauda*** |
|  | Black-fronted Tern | *Chlidonias albostriata* |
|  | **Black-naped Tern** | ***Sterna sumatrana*** |
|  | Blue Noddy | *Procelsterna cerulea* |
|  | **Bridled Tern** | ***Sterna anaethetus*** |
|  | **Brown Noddy** | ***Anous stolidus*** |
|  | **Caspian Tern** | ***Sterna caspia*** |
|  | **Cayenne Tern** | ***Sterna eurygnatha*** |
|  | Chinese Crested tern | *Sterna bernsteini* |
|  | **Common Tern** | ***Sterna hirundo*** |
|  | **Crested Tern** | ***Sterna bergii*** |
|  | **Damara Tern** | ***Sterna balaenarum*** |
|  | Elegant Tern | *Sterna elegans* |
|  | Fairy Tern | *Sterna nereis* |
|  | Forster's Tern | *Sterna forsteri* |
|  | Gray Noddy | *Procelsterna albivitta* |
|  | Gray-backed Tern | *Sterna lunata* |
|  | **Gull-billed Tern** | ***Sterna nilotica*** |
|  | Inca Tern | *Larosterna inca* |
|  | Kerguelen Tern | *Sterna virgata* |
|  | **Least Tern** | ***Sterna antillarum*** |
|  | **Lesser Crested Tern** | ***Sterna bengalensis*** |
|  | **Lesser Noddy** | ***Anous tenuirostris*** |
|  | **Lesser White Tern** | ***Gygis microrhyncha*** |
|  | **Little Tern** | ***Sterna albifrons*** |
|  | Peruvian Tern | *Sterna lorata* |
|  | **Roseate Tern** | ***Sterna dougallii*** |
|  | **Royal Tern** | ***Sterna maxima*** |
|  | **Sandwich Tern** | ***Sterna sandvicensis*** |
|  | Saunder's Tern | *Sterna saundersi* |
|  | South American Tern | *Sterna hirundinacea* |
|  | White Tern | *Gygis alba* |
|  | White-cheeked Tern | *Sterna repressa* |
|  | **White-fronted Tern** | ***Sterna striata*** |
| Alcidae | **Dovekie** | ***Alle alle*** |
|  | Ancient Murrelet | *Synthliboramphus antiquus* |
|  | **Atlantic Puffin** | ***Fratercula arctica*** |
|  | **Black Guillemot** | ***Cepphus grylle*** |
|  | **Cassin's Auklet** | ***Ptychoramphus aleuticus*** |
|  | **Common Murre** | ***Uria aalge*** |
|  | Craveri's Murrelet | *Endomychura craveri* |
|  | Crested Auklet | *Aethia cristatella* |
|  | **Horned Puffin** | ***Fratercula corniculata*** |
|  | Japanese Murrelet | *Synthliboramphus wumizusume* |
|  | Kittlitz's Murrelet | *Brachyramphus brevirostris* |
|  | Least Auklet | *Aethia pusilla* |
|  | Long-billed Murrelet | *Brachyramphus perdix* |
|  | **Marbled Murrelet** | ***Brachyramphus marmoratus*** |
|  | Parakeet Auklet | *Cyclorrhynchus psittacula* |
|  | **Pigeon Guillemot** | ***Cepphus columba*** |
|  | **Razorbill** | ***Alca torda*** |
|  | **Rhinoceros Auklet** | ***Cerorhinca monocerata*** |
|  | Spectacled Guillemot | *Cepphus carbo* |
|  | **Thick-billed Murre** | ***Uria lomvia*** |
|  | **Tufted Puffin** | ***Fratercula cirrhata*** |
|  | Whiskered Auklet | *Aethia pygmaea* |
|  | Xantus' Murrelet | *Endomychura hypoleuca* |
