# Supplementary material for: Population Trend of the World’s Monitored Seabirds, 1950-2010
Source: PLoS One. 2015 Jun 9;10(6):e0129342. doi: 10.1371/journal.pone.0129342 (PMC4461279; doi:10.1371/journal.pone.0129342)
Supplement: S2 Table — (DOCX) [file pone.0129342.s002.docx]

Supplementary online materials to

Population trend of the world’s monitored seabirds, 1950-2010

by

Michelle Paleczny^1¶^*, Edd Hammill^1,2¶^,Vasiliki Karpouzi^1^, Daniel Pauly^1^

^1^ University of British Columbia, Vancouver, British Columbia, Canada

^2^ School of the Environment, University of Technology, Sydney, Ultimo, New South Wales 2007, Australia

¶ The first and second authors contributed equally to this work.

*Correspondence to: e-mail: m.paleczny@fisheries.ubc.ca

**S2 Table. List of coastal stretches for which breeding population data were available.**

| Chukchi Sea | Beaufort Sea | Northwest Territories | East Siberian Sea | Laptev Sea |
| --- | --- | --- | --- | --- |
| Kara Sea | Hudson Bay | Digges Island | Coats Island | Southampton Island |
| Queen Elizabeth Islands | Greenland North | Coburg Island | Bylot Island | Prince Leopold Island |
| Banks Island | Baffin Island W | Victoria Island | Baffin Island E | Baffin Island SE |
| NE US | Scotian Shelf | Quebec | Newfoundland-Labrador | Nain |
| Greenland West | Baffin Bay | Ellesmere Island SE | Bear Island | Jan Mayen Island |
| Svalbard | Franz Josef Land | Azores | Greenland East | Barents Sea |
| White Sea | Novaya Zemlya | Norwegian Shelf N | Norwegian Shelf SW | Norwegian Sea |
| Belgium | Holland | England E | Scotland E | Shetland Islands |
| Germany | Denmark | Channel Islands | Baltic Sea | Sweden |
| Finland | Estonia | Latvia | Lithuania | Poland |
| Scotland W | France | Wales | Ireland | England W |
| Portugal | Berlenga Islands | Iberian | Iceland | Faeroes |
| Bermuda Islands | Gulf of Mexico | Florida | Mexico Gulf | SE US |
| Caribbean | Costa Rica Gulf | Trinidad | Puerto Rico | Bahamas |
| Haiti | Cuba | Jamaica | Cayman Islands | US Virgin Islands |
| Br Virgin Islands | Antigua Barbuda | Netherlands Antilles | Montserrat | Guadeloupe |
| Dominica | Martinique | Anguilla | Belize | Mexico Caribbean |
| Grenada | Dominican Republic | Navassa Island | Venezuela | Colombia |
| Turks Caicos | Honduras Caribbean | Aruba | Barbados | St Paul |
| Cape Verde | Salvages | Madeira | Canary | Senegal |
| Gambia | Mauritania | Morocco | Guinea-Bissau | Guinea |
| Ghana | Benin | Cameroon | Gabon | Sao Tome Principe |
| Chafarinas Islands | Spain SE | Spain E | Spain NE | Balearic Islands |
| France MED | Corsica | Sardinia | Morocco MED | Algeria |
| Tunisia | Italy W | Italy NE | Sicily | Slovenia |
| Croatia | Albania | Greece W | Greece N | Greece S |
| Libya | Malta | Egypt MED | Turkey W | Turkey S |
| Cyprus | Lebanon | Israel | Serbia Montenegro | Russia S |
| Romania | Sivash | Dniester | Dnieper | AzovUKR |
| Bulgaria | AzovRUS | Turkey N | Marmara Sea | Georgia |
| Trindade and Martin Vaz Archipelago | Diego Ramirez Islands | Argentina | Falkland Islands | Tierra del Fuego |
| Uruguay | South Brazil | Brazil East | Noronha Archipelago | Rio Grande do Norte |
| North Brazil | French Guiana | Ascension Island | St Helena | Gough Islands |
| Angola | Namibia | South Africa West | South Africa East | South Georgia |
| South Orkney Islands | South Sandwich Islands | Bouvet Island | South Shetland Islands | Antarctic Peninsula |
| Weddell Sea | Dronning Maud Land West | Reunion Island | Mauritious | Seychelles |
| Amsterdam Island | Chagos | Mozambique | Europa Island | Madagascar |
| Juan de Nova | Glorieuses | Comoros | Tanzania | Kenya |
| Somalia | Persian Gulf | Oman | India W | Bahrain |
| Yemen | Maldives | Pakistan | Socotra | Djibouti |
| Qatar | Kuwait | IranPERS | United Arab Emirates | Saudi Arabia PERS |
| India E | Bangladesh | Myanmar | Eritrea | Sudan |
| Egypt | Saudi Arabia | Cocos Islands | Christmas Island | Sri Lanka |
| Tasmania | Albatross Island | S Australia | W Australia | Houtman Abrolhos |
| Penguin Island | SW Australia | Adele Island | Ashmore Reef | Prince Edward Islands |
| Crozet Islands | Kerguelen Islands | McDonald Islands | Dronning Maud Land East | Adelie Land |
| Enderby Land | Queen Mary Land | Wilkes Land | George V Land | Mac. Robertson Land |
| Bonin Islands | Izu Islands | Hong Kong | Taiwan | Viet Nam N |
| Ryuku Islands | Shanghai | Matsu Islands | Yellow Sea | Liaodong |
| Yancheng | South Korea | N Korea W | Kyushu | Honshu |
| Japan | Vladivostok | Teuri Island | Hokkaido | Kurile |
| Okhotsk Sea N | Sakhalin Island | Okhotsk Sea | West Bering Sea | Commander Islands |
| Kamchatka E | East Bering Sea | Pribilof Islands | Aleutian Chain | Buldir Islands |
| St Matthew Island | Gulf of Alaska | British Columbia | Alaska SE | Vancouver Is W |
| Alaska Peninsula | Graham Is W | Moresby Is W | Moresby Is E | BC N coast |
| Scott Islands | Queen Charlotte Strait | Georgia Strait | Gulf Islands | Washington State |
| Oregon State | Mariana Islands | Caroline Islands | Marshall Islands | New Caledonia |
| Solomon Islands | Fiji | Kiribati | Nauru | Thailand |
| Cambodia | Malaysia East | Sabah | Philippines | Banda Sea |
| Indonesian Sea | North Australia | Queensland | Great Barrier Reef | French Polynesia |
| Line Islands | Phoenix Islands | Tonga | Cook Islands | Tokelau |
| Kermadec | American Samoa | Samoa | Johnston Atoll | California State |
| California Bight | Islands off Mexico | Baja California | Sea of Cortes | Hawaii |
| Mexico West | Costa Rica Pacific | El Salvador | Nicaragua Pacific | Panama Pacific |
| Macquarie Island | Auckland Island | Campbell Island | Antipodes Islands | Bounty Islands |
| Chatham Islands | Norfolk Island | Pitcairn Islands | New South Wales | Lord Howe Island |
| Victoria | New Zealand | Snares Islands | Solander Island | Three Kings Islands |
| Sala y Gomez Islands | Easter Island | Juan Fernandez Islands | Galapagos | San Felix |
| Malpelo Island | Ecuador | La Plata Island | Peru | Chile N |
| Chile S | Victoria Land | Ross Sea | Balleny Islands | Amundsen Sea |
| Bellingshausen Sea | Peter Island |  |  |  |
