# Supplementary material for: Population Trend of the World’s Monitored Seabirds, 1950-2010
Source: PLoS One. 2015 Jun 9;10(6):e0129342. doi: 10.1371/journal.pone.0129342 (PMC4461279; doi:10.1371/journal.pone.0129342)
Supplement: S3 Table — (DOCX) [file pone.0129342.s003.docx]

Supplementary online materials to

Population trend of the world’s monitored seabirds, 1950-2010

by

Michelle Paleczny^1¶^*, Edd Hammill^1,2¶^,Vasiliki Karpouzi^1^, Daniel Pauly^1^

^1^ University of British Columbia, Vancouver, British Columbia, Canada

^2^ School of the Environment, University of Technology, Sydney, Ultimo, New South Wales 2007, Australia

¶ The first and second authors contributed equally to this work.

*Correspondence to: e-mail: m.paleczny@fisheries.ubc.ca

**S3 Table. Example of contents of the population size database for the Blue Petrel (*Halobaena caerulea*).**

| **Species** | **Coastal stretch** | **Year** | **Number of individuals** | **References** |
| --- | --- | --- | --- | --- |
| Blue Petrel | Crozet Islands | 1982 | 207,900 | Jouventin *et al.* 1984 |
| Blue Petrel | Crozet Islands | 1984 | 294,015 | www.birdlife.net/datazone |
| Blue Petrel | Diego Ramirez Islands | 1982 | 2,000,000 | Fugler *et al.* 1987 |
| Blue Petrel | Diego Ramirez Islands | 2002 | 3,105,000 | Lawton *et al*. 2006 |
| Blue Petrel | Kerguelen Islands | 1985 | 466,690 | Weimerskirch *et al.* 1989 |
| Blue Petrel | Kerguelen Islands | 1989 | 495,000 | www.birdlife.net/datazone |
| Blue Petrel | Kerguelen Islands | 2002 | 1,866,762 | Barbraud & Delord 2006 |
| Blue Petrel | Macquarie Island | 1979 | 1,980 | Rounsevell & Brothers 1984 |
| Blue Petrel | Macquarie Island | 1984 | 1,815 | Brooke 2004 |
| Blue Petrel | Prince Edward Islands | 1977 | 99,000 | Williams *et al.* 1979 |
| Blue Petrel | Prince Edward Islands | 1982 | 99,000 | Williams 1984 |
| Blue Petrel | Prince Edward Islands | 1990 | 1,815,000 | Cooper & Brown 1990 |
| Blue Petrel | Prince Edward Islands | 1997 | 660,000 | www.birdlife.net/datazone |
| Blue Petrel | South Georgia | 1980 | 231,000 | Croxall *et al.* 1985 |
| Blue Petrel | South Georgia | 1982 | 231,000 | Croxall *et al.* 1984 |
